# Hydrogen sulphide exacerbates acute pancreatitis by over‐activating autophagy *via* AMPK/mTOR pathway

**DOI:** 10.1111/jcmm.12928

**Published:** 2016-07-15

**Authors:** Liang Ji, Le Li, Fengzhi Qu, Guangquan Zhang, Yongwei Wang, Xuewei Bai, Shangha Pan, Dongbo Xue, Gang Wang, Bei Sun

**Affiliations:** ^1^ Department of General SurgeryThe First Affiliated Hospital of Harbin Medical UniversityHarbinChina; ^2^ Central LaboratoryThe First Affiliated Hospital of Harbin Medical UniversityHarbinChina

**Keywords:** acute pancreatitis, hydrogen sulphide, impaired autophagy, AMPK, mTOR

## Abstract

Previously, we have shown that hydrogen sulphide (H_2_S) might be pro‐inflammatory during acute pancreatitis (AP) through inhibiting apoptosis and subsequently favouring a predominance of necrosis over apoptosis. In this study, we sought to investigate the detrimental effects of H_2_S during AP specifically with regard to its regulation on the impaired autophagy. The incubated levels of H_2_S were artificially intervened by an administration of sodium hydrosulphide (NaHS) or DL‐propargylglycine (PAG) after AP induction. Accumulation of autophagic vacuoles and pre‐mature activation of trypsinogen within acini, which indicate the impairment of autophagy during AP, were both exacerbated by treatment with NaHS but attenuated by treatment with PAG. The regulation that H_2_S exerted on the impaired autophagy during AP was further attributed to over‐activation of autophagy rather than hampered autophagosome–lysosome fusion. To elucidate the molecular mechanism that underlies H_2_S‐mediated over‐activation of autophagy during AP, we evaluated phosphorylations of AMP‐activated protein kinase (AMPK), AKT and mammalian target of rapamycin (mTOR). Furthermore, Compound C (CC) was introduced to determine the involvement of mTOR signalling by evaluating phosphorylations of downstream effecters including p70 S6 kinase (P70S6k) and UNC‐51‐Like kinase 1 (ULK1). Our findings suggested that H_2_S exacerbated taurocholate‐induced AP by over‐activating autophagy *via* activation of AMPK and subsequently, inhibition of mTOR. Thus, an active suppression of H_2_S to restore over‐activated autophagy might be a promising therapeutic approach against AP‐related injuries.

## Introduction

Acute pancreatitis (AP) is an abdominal emergency with considerable morbidity and mortality because of its concomitant organ failures and other eventful complications 1. Although the exact mechanisms involved in the pathogenesis of AP have not been well elucidated, accumulation of cytoplasmic vacuoles and pre‐mature activation of trypsinogen within acini were long noticed as the two early‐phase features of AP. Recently, in addition to that the accumulated vacuoles were defined as autophagic in origin, the pre‐mature activation of trypsinogen was shown to be a consequence of the impaired autophagy 2, 3, 4, 5.

Macroautophagy (henceforth, ‘autophagy’) is a cellular protective and quality control process which serves as a salvage mechanism for recycling cytoplasmic materials and preserving energy *via* lysosome‐driven degradations in response to a variety of extracellular and intracellular stresses including nutrients deprivation, hormonal therapy, pathogenic infection, misfolded proteins and damaged organelles 6. The mechanisms by which autophagy is induced are not fully understood. Fundamental or physiological autophagy is an essential cellular self‐aid behaviour in harsh environment and beyond this range it will lead to cell damage. Impaired autophagy is considered as a major contributor to many neurodegenerative disorders, cardiovascular diseases, inflammations and ischaemia/reperfusion injuries 7, 8, 9, 10, 11. The execution of autophagy involves a set of evolutionarily conserved gene products, known as Atg proteins. Among these Atg proteins, microtubule‐associated protein 1 light chain 3 (LC3, the mammalian homologue of the yeast Atg6) serves as a widely used marker of autophagosomes. LC3 conversion (LC3‐II/I) usually correlates well with the accumulation of autophagosomes 6.

Hydrogen sulphide (H_2_S) is the third most common endogenously produced gaseous signalling molecule that is synthesized from L‐cysteine primarily by two key enzymes: cystathionine‐γ‐lyase (CSE) in the peripheral tissues and cystathionine‐β‐synthetase (CBS) in the central nervous system. H_2_S is considered to be pivotal in human physiology despite its toxic nature known for centuries. In addition to its pathophysiological relevance in cardiovascular and neuronal disorders, accumulating focus has been placed on the involvement of H_2_S in inflammatory diseases. Generally, H_2_S is believed to be increasingly synthesized and to play a pro‐inflammatory role during AP 12, 13, 14, 15, 16, but the mechanisms involved remain obscure and controversial. Two thirds of H_2_S molecules dissociate into hydrogen ions and hydrosulphide ions under physiological conditions, sodium hydrosulphide (NaHS) herein can be administrated as an exogenous water‐soluble H_2_S donor 15, 16. In contrast, DL‐propargylglycine (PAG) is usually administered to block endogenous H_2_S synthesis by irreversibly inhibiting CSE 12, 15.

Previously, we have shown that inhibition of H_2_S synthesis effectively alleviated the necrotic injuries in AP rats by significantly promoting acini apoptosis 14. To further investigate the relevance of H_2_S in AP, the effects of H_2_S on impaired autophagy and the possible mechanisms that account for them were evaluated in the present study.

## Materials and methods

### Reagents

All chemicals were purchased from Sigma‐Aldrich (St. Louis, MO, USA) unless stated otherwise. The primary antibodies used for immunoblot were: Beclin‐1 (1:1000), p62 (1:1000), LC3 (1:1000), mammalian target of rapamycin (mTOR, 1:1000), p‐mTOR (Ser2448; 1:1000), AKT (1:1000), p‐AKT (Ser473; 1:1000), AMP‐activated protein kinase (AMPK, 1:1000), p‐AMPK (Thr172; 1:1000), p70 S6 kinase (P70S6K, 1:1000), p‐P70S6K (Thr389; 1:1000), UNC‐51‐like kinase 1 (ULK1, 1:1000), p‐ULK1 (Ser757; 1:1000) and high mobility group protein B 1 (HMGB1, 1:1000) purchased from Cell Signaling Technology (Danvers, MA, USA), and lysosome‐associated membrane protein‐2 (LAMP‐2, 1:500) purchased from Thermo Fisher Scientific (Rockford, IL, USA). The primary antibodies used for immunohistochemistry (IHC) and immunofluorescence (IF) were LC3 (1:200 for IHC and 1:25 for IF) purchased from Sigma‐Aldrich and LAMP‐2 (1:200 for IHC and 1:50 for IF) purchased from Thermo Fisher Scientific.

### Animals and ethics statement

One hundred and twenty male Wistar rats, weighing 200–250 g, were supplied by the Animal Research Center at the First Clinical College of Harbin Medical University (Harbin, China). The rats were fed with rodent chow and water *ad libitum* in an environmentally controlled room (18–21°C, 40–60% relative humidity, 12‐hr light/dark cycle). After 1 week of acclimatization, the rats were deprived of food overnight before the experiments. The animal care and experimental protocols were all approved by the Institutional Animal Care and Use Committee of Harbin Medical University (no.: 2015005) and also conducted in accordance with the Guide for the Care and Use of Laboratory Animals.

### Model establishment and experimental design *in vivo*

A model of AP in rats was established using the method as previously described 14, 17, 18, 19, 20. Briefly, the rats were anaesthetized by an intra‐peritoneal injection of sodium pentobarbital (40 mg/kg). Acute pancreatitis was induced by a retrograde infusion of 3.5% sodium taurocholate (Na‐TC, 0.15 ml/100 g) into the pancreaticobiliary duct. The rats were randomly allocated into four groups: AP group (*n* = 30, performed the operation as mentioned above), NaHS group [*n* = 30, administered an intra‐peritoneal injection of a NaHS solution (1 ml; 28 μmol/kg) 1 hr after AP induction], PAG group [*n* = 30, administered an intra‐peritoneal injection of a PAG solution (1 ml; 80 mg/kg) 1 hr after AP induction] and sham group (*n* = 30, underwent a midline laparotomy and separation of pancreaticobiliary duct merely). The dosages were selected on the basis of previous reports 14, 21, 22 and our preliminary experiments. All of the surviving rats in each group were randomly killed at 3, 6 and 12 hrs after AP induction. For every single blood sample, the serum was obtained after a centrifugation at 876 × g for 15 min. and then stored at −80°C until assayed. For every single pancreatic sample, three segments were prepared for different purposes: rinsed in saline buffer and snap‐frozen in liquid nitrogen at −80°C for immunoblot, fixed in 4% buffered paraformaldehyde for 48 hrs and then embedded in paraffin for haematoxylin and eosin staining, IHC and IF, and fixed in 2 ml of 2.5% glutaraldehyde and post‐fixed in 1% osmium tetroxide solution for transmission electron microscopy (TEM).

### Measurement of serum H_2_S

The serum H_2_S level was measured as previously described 14, 22. In brief, aliquots (75 μl) of sera were mixed with 100 μl of distilled water and 300 μl of 10% trichloroacetic acid. The reaction was stopped by the addition of 150 μl of 1% zinc acetate. Then, *N*,* N*‐dimethyl‐*p*‐phenylenediamine sulphate (20 μM; 100 μl) in 7.2 M HCl and FeCl_3_ (30 μM; 133 μl) in 1.2 M HCl were added. After incubation for 15 min., the absorbance of the resulting solution at 670 nm was measured with a spectrophotometer (UV‐2550; Shimadzu, Kyoto, Japan). All samples were assayed in triplicate, and H_2_S was calculated against a calibration curve of NaHS (0.122–250 μM).

### Measurement of H_2_S synthesizing activity in pancreas

Hydrogen sulphide synthesizing activity in pancreatic homogenates was measured as previously described 14, 22. Briefly, pancreatic samples were homogenized in 50 mM of ice‐cold potassium phosphate buffer (pH 6.8) containing 100 mM of potassium phosphate buffer, 10 mM of L‐cysteine and 2 mM of pyridoxal 5′‐phosphate. The reaction was performed in cryovial test tubes and initiated by transferring tubes from ice to a shaking water bath at 37 °C. After incubation for 30 min., 250 μl of 1% zinc acetate was added to trap evolved H_2_S, and this was followed by 250 μl of 10% trichloroacetic acid to denature the protein and stop the reaction. Subsequently, *N*,* N*‐dimethyl‐*p*‐phenylenediamine sulphate (20 μM; 133 μl) in 7.2 M HCl was added, and this was immediately followed by FeCl_3_ (30 μM; 133 μl) in 1.2 M HCl. The absorbance of the resulting solution at 670 nm was measured. The H_2_S concentration was calculated against a calibration curve of NaHS. Results were expressed as nanomoles of NaHS equivalent formed per milligram of tissue.

### Measurement of parameters

The pancreatic levels of interleukin 1β (IL‐1β), tumour necrosis factor‐α (TNF‐α), monocyte chemotactic protein 1 (MCP‐1) and macrophage inflammatory protein 1α (MIP‐1α) were measured using ELISA kits (R&D Systems, Minneapolis, MN, USA) according to the manufacturer's instructions 14. The serum levels of amylase and lipase were spectrophotometrically measured with an autobiochemical analyser (Toshiba, Tokyo, Japan) as previously described 17.

### Haematoxylin and eosin staining

Haematoxylin and eosin staining was performed to observe the level of inflammation and tissue damage under a light microscope (4×). Two professional pathologists who were blinded to the experimental protocol scored the pancreatic tissue on a scale from 0 to 4 for the degrees of edema, inflammation, haemorrhage and necrosis in 20 randomly selected fields. We applied the scoring system defined by Kusshe *et al*. 23 and the final scores of each histopathological examination were totalled.

### Immunohistochemistry

The protocol for IHC has been previously described 14, 17. In short, the specimens were dewaxed and incubated with 3% H_2_O_2_ in methanol at 37 °C for 10 min. to quench endogenous peroxidase. After blockage at room temperature for 30 min., the sections were incubated with specific primary antibodies overnight at 4°C. Subsequently, the sections were incubated with secondary antibodies (1: 200; ZSGB‐BIO, Beijing, China) and developed for colour with diaminobenzidine peroxidase colour development kits (ZSGB‐BIO). Finally, the sections were counterstained with haematoxylin. The sections were observed under a light microscope and the expression of protein was quantified by integrated optical density (IOD) with Image‐Pro Plus v6.0 software (Media Cybernetics, Crofton, MA, USA) in 20 randomly selected fields. The cells with the presence of a dark reddish‐brown chromogen indicate the positive signal.

### Immunocolocalization assay between LC3 and LAMP‐2 in pancreas

To examine autophagosome–lysosome fusion, an immunocolocalization assay between LC3 (an autophagosome membrane marker) and LAMP‐2 (a lysosome membrane marker) in pancreas was conducted. Paraffin‐embedded pancreatic tissue sections (5 μm) were dewaxed, rehydrated and incubated with 5% H_2_O_2_ in methanol for 10 min. to quench the endogenous peroxidase. After high‐pressure antigen retrieval, the sections were blocked with 5% bovine serum albumin for 1 hr and incubated with the primary antibodies overnight at 4°C. On the following day, secondary goat antimouse Alexa Fluor^®^ 488 (1:100) and goat antirabbit Alexa Fluor^®^ 647 (1:100) antibodies (Abcam, Cambridge, UK) were applied for 1 hr. Finally, the sections were covered with mounting medium (Vector Laboratories, Burlingame, CA, USA) and observed under a confocal laser scanning microscope (LSM‐510; Carl Zeiss, Oberkochen, Germany). With the ImageJ 1.48v software (National Institutes of Health, Bethesda, MD, USA), the percentage of LAMP‐2 stained area that colocalized with LC3 [yellow area/(yellow area + free red area) × 100%] per high power field in the merged image was calculated.

### Transmission electron microscopy

The fixed samples were dehydrated through a graded series of ethanol and embedded in epoxy resin. Ultrathin sections (80 nm) were collected on copper grids, double‐stained with uranyl acetate and lead citrate, and then examined under a Hitachi H‐7100 transmission electron microscope (Hitachinaka, Japan) at 80 kV. For quantifications, the percentage of autophagic vacuoles per cytoplasm area was calculated on each print.

### Cell cultures

The rat pancreatic exocrine cell line AR42J was purchased from the American Type Culture Collection (Manassas, VA, USA) and cultured in Dulbecco's modified Eagle's medium (Gibco, Grand Island, NY, USA) supplemented with 10% foetal bovine serum (ScienCell, San Diego, CA, USA), 100 U/ml of penicillin and 100 mg/ml of streptomycin (Invitrogen, Carlsbad, CA, USA) at 37°C in a 5% CO_2_ humidified incubator.

### Experimental design *in vitro*

To stimulate AP *in vitro*, AR42J cells were treated with 500 μM of Na‐TC based on previous reports 24, 25, 26 and our preliminary experiments. For control group, the cells were treated with an equivalent volume of PBS to that of Na‐TC administrated in AP group. For NaHS or PAG group, a treatment with NaHS (100 μM) for 30 min. or PAG (3 mM) for 60 min. before AP induction was administered 16, 27, 28. Alternatively, the cells were incubated with 10 μM of chloroquine (CQ) for 2 hrs or 20 μM of Compound C (CC) for 1 hr before the experiments 29, 30, 31, 32. The cell viability was measured with a Cell Counting Kit‐8 (Dojindo Molecular Technologies, Kumamoto, Japan) and no significant changes were observed upon the aforementioned treatments.

### Autophagy flux assay

To facilitate the autophagy flux assay, the AR42J cells were transfected with a mRFP‐GFP‐LC3 tandem lentivirus (Genechem Co., Shanghai, China) according to the manufacturer's instructions. Theoretically, GFP is a stably folded protein and relatively resistant to lysosomal proteases. However, the low pH level inside the lysosomes quenches the fluorescent signal of GFP. With this construct, autophagosomes and autolysosomes were labelled with yellow (mRFP and GFP) and red (mRFP only). After treatments, the images of differently allocated cells were captured using a confocal laser scanning microscope (LSM‐510; Carl Zeiss) and analysed using the Image‐Pro Plus v6.0 software. We calculated the ratio of autolysosomes (red) to autophagosomes (yellow) per cell to evaluate the extent of autophagosomes maturation into autolysosomes.

### Trypsinogen activation assay

The trypsinogen activation assay was performed as previously described 33 with minor modifications directed by our preliminary experiments. In brief, after an equilibration for 30 min., 500 μM of Na‐TC was administered for up to 40 min. with or without pre‐condition of NaHS or PAG. The AR42J cells were then washed, resuspended in NaHEPES solution without Na‐TC and supplemented with 10 μM of (CBZ‐Ile‐Pro‐Arg)_2_‐rhodamine 110 (BZiPAR; Invitrogen), a specific substrate for trypsin that becomes fluorescent after cleavage of the two oligopeptide side chains. To exclude the false positive observations derived from the trypsin released into the extracellular fluid, soybean trypsin inhibitor (5 mM) was introduced. BZiPAR was excited with a 488 nm laser line and emission was collected in the 505–530 nm band for cleaved BZiPAR. The images were captured using a confocal laser scanning microscope (LSM‐510; Carl Zeiss) and analysed using the Image‐Pro Plus v6.0 software. The percentage of positive cells was calculated to indicate the extent of trypsinogen activation.

### Immunoblot

The immunoblot techniques have been previously described 14, 17. In brief, pancreatic tissues or cells were homogenized in protein lysate buffer that contained protease inhibitor and phosphatase inhibitor (Roche, Shanghai, China), and debris was removed by centrifugation. The samples were resolved on polyacrylamide sodium dodecyl sulphate gels and electrophoretically transferred to polyvinylidene difluoride membranes. The membranes were blocked with 5% skimmed milk, incubated with the appropriate primary antibodies and horseradish peroxidase‐conjugated secondary antibodies (1:2000; ZSGB‐BIO). Immunostained bands were detected using enhanced chemiluminescence kits (Pierce Chemical, Rockford, IL, USA). β‐actin (1:2000; Santa Cruz Biotechnology, Santa Cruz, CA, USA) was used as the protein loading control and the level of protein expression was calibrated as the relative band density to that of β‐actin.

### Statistical analysis

Data were presented as mean ± S.D. of three independent experiments and analysed using SAS 9.1 for Windows (SAS Institute, Cary, NC, USA). The data were analysed using a one‐way anova followed by a Scheffe test. A *P*‐value of <0.05 was taken to be statistically significant.

## Results

### AP‐related injuries in rats positively correlated with the incubated level of H_2_S

**Figure 1:**
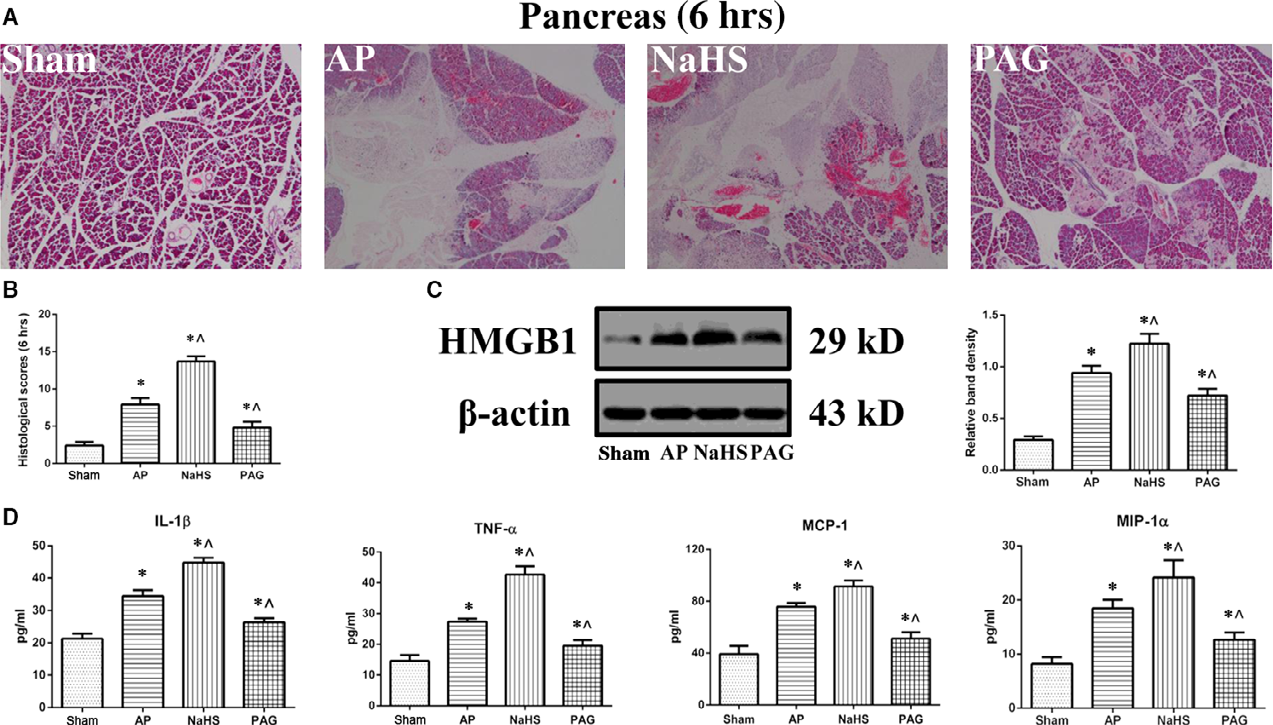
Acute pancreatitis (AP)‐related injuries in rats positively correlated with the incubated level of H_2_S. (**A**) Representative photographs (4 × ) of haematoxylin and eosin‐stained pancreatic tissues harvested from the rats that were subjected to sham operation, AP, AP+NaHS or AP+PAG for 6 hrs since AP induction. (**B**) Histopathological scoring was performed to evaluate the pancreatic injuries of the rats as described above. (**C**) Representative immunoblot images (left) and quantitations (right) of HMGB1 expression in pancreatic tissues harvested from the rats as described above. β‐actin was used as the protein loading control. (**D**) The levels of IL‐1β, TNF‐α, MCP‐1 and MIP‐1α (left to right) in pancreatic tissues harvested from the rats, as described above, were assessed by ELISA. Data were presented as mean ± S.D. (*n* = 3). *P < 0.05 *versus* sham, ^*P* < 0.05 *versus* AP.

The pathomorphological alterations of pancreatic tissues were observed by haematoxylin and eosin staining at 3, 6 and 12 hrs after AP induction or sham operation (Fig. 1A and B, Fig. S1A–D). The attack of AP was associated with various levels of oedema, necrosis, inflammatory cell infiltration and haemorrhage, whereas there was no evident ectopic alterations in rats that underwent a sham operation. Histopathological scoring was introduced to quantify these pathomorphological alterations. After AP induction, the histological score was significantly increased compared with that in sham group at 3, 6 and 12 hrs (Fig. 1B, Fig. S1C and D). Furthermore, the administration of NaHS significantly elevated the histological score, whereas the administration of PAG significantly attenuated the score at 6 hrs since AP induction, compared with that in AP group (Fig. 1B). However, such statistically significant deviations that correlated with the interventions against the incubated level of H_2_S could not be satisfactorily identified at 3 or 12 hrs (Fig. S1C and D). Given that the aim was to determine the role of H_2_S during AP, 6 hrs was chosen for subsequent observations *in vivo*. To confirm the increase in the level of H_2_S after AP induction and the validation of our interventions, the serum levels of H_2_S and H_2_S synthesizing activities in pancreatic homogenates were calculated against a calibration curve of NaHS and expressed as NaHS equivalent. There was a positive correlation between the incubated level of H_2_S and histological score. The level of HMGB1, a well‐known member of damage‐associated molecular patterns released from the nuclei of necrotic cells 18, 23, was detected by immunoblot (Fig. 1C). In accordance with aforementioned results, AP induction significantly increased the level of HMGB1. Administration of NaHS significantly augmented the level of HMGB1, whereas the administration of PAG significantly decreased the level of HMGB1, compared with that in AP group. In addition, some cytokines were measured to evaluate the inflammatory status within pancreatic homogenates. Figure 1D lists the pancreatic levels of IL‐1β, TNF‐α, MCP‐1 and MIP‐1α among groups and confirms aforementioned findings with statistical significance. That is, the increased level of H_2_S is correlated with the increasing concentration of pancreatic cytokines and *vise versa*. To sum up, the level of H_2_S was increased in Na‐TC‐induced AP rats and positively correlated with disease severity.

### Accumulation of autophagic vacuoles positively correlated with the incubated level of H_2_S

**Figure 2:**
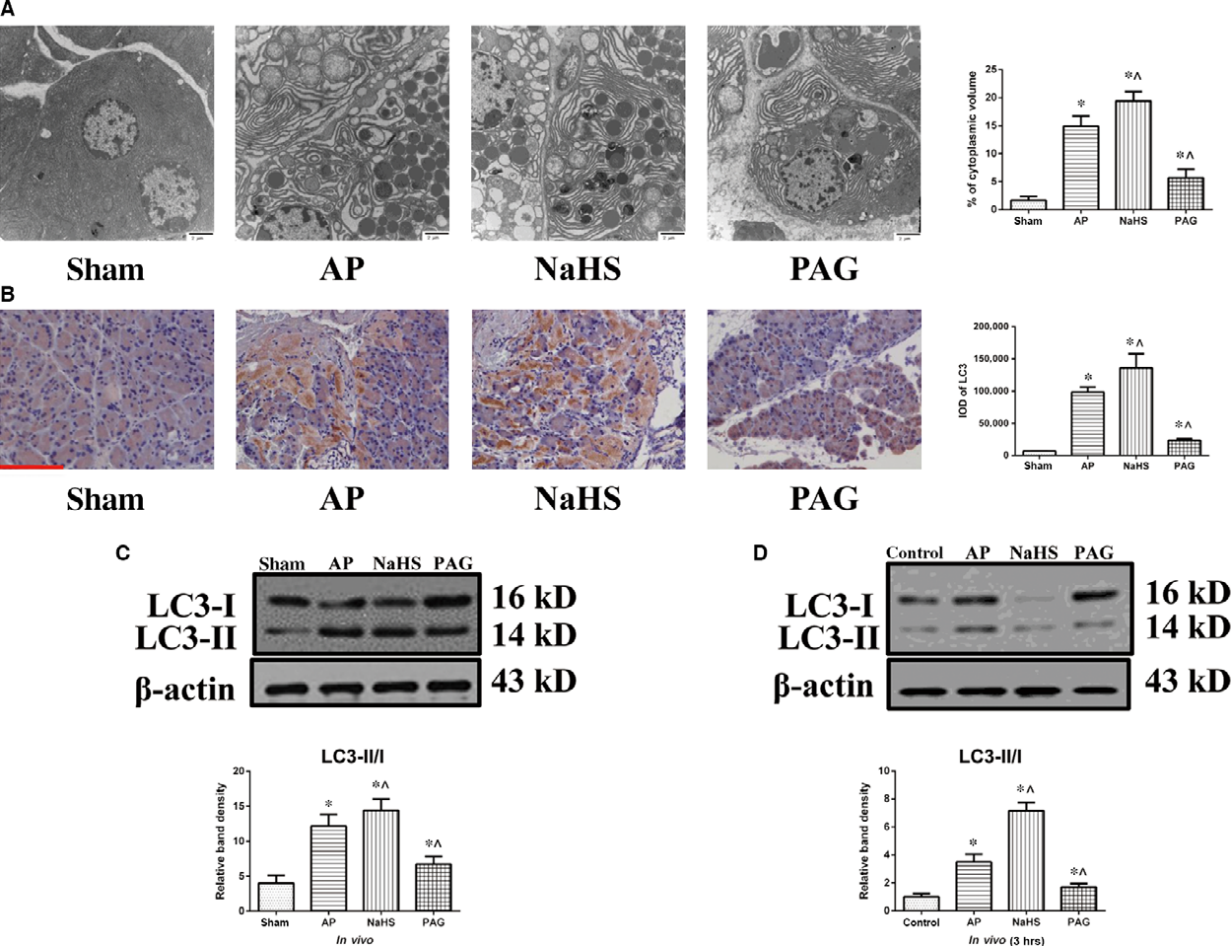
Accumulation of autophagic vacuoles positively correlated with the incubated level of H_2_S. (**A**) Representative TEM photographs of pancreatic tissues harvested from the rats as described in Figure 1A. The percentage of autophagic vacuoles per cytoplasm area was totalled, bar: 2 μm. (**B**) Representative IHC photographs and corresponding IODs of LC3 expression in pancreatic tissues harvested from the rats as described in Figure 1A, bar: 50 μm. (**C** and **D**) Representative immunoblot images of LC3 expression (top) and quantitations of LC3 conversion (bottom) in pancreatic tissues (**C**) harvested from the rats as described in Figure 1A and in AR42J cells (**D**) that were subjected to control, AP, AP+NaHS or AP+PAG for 3 hrs since AP induction. β‐actin was used as the protein loading control. Data were presented as mean ± S.D. (*n* = 3). *P < 0.05 *versus* sham or control, ^*P* < 0.05 *versus* AP.

Transmission electron microscopy revealed that, in addition to the structural disturbance in endoplasmic reticulum and mitochondria, there were also some autophagic vacuoles (the two‐layer membrane structures that wrapped around the partially degraded cargos) in the sections of experimental groups. In contrast, there was no evident abnormality in sham group. The percentage of autophagic vacuoles per cytoplasm area was significantly increased after AP induction, compared with that in sham group. Moreover, the administration of NaHS significantly potentiated the percentage of autophagic vacuoles per cytoplasm area, whereas the administration of PAG significantly weakened the percentage of autophagic vacuoles per cytoplasm area, compared with that in AP group (Fig. 2A). The LC3 expression was detected using IHC. Compared with its very weak cytoplasmic expressions in sham group, there were moderate to strong cytoplasmic LC3 expressions and coarse punctuated signallings (represent the autophagosome membrane‐bound form of LC3, LC3‐II) in experimental groups. The IOD measurements indicated that there was a significant increase in LC3 expression in experimental groups, compared with that in sham group. The LC3 expression was significantly enhanced by the administration of NaHS compared with that caused by AP induction alone, whereas the administration of PAG significantly attenuated the LC3 expression caused by AP induction alone (Fig. 2B). In addition, LC3 conversion was evaluated by immunoblot to assess the accumulation of autophagosomes both *in vivo* and *in vitro*. *In vivo* (Fig. 2C), LC3 conversion was significantly elevated in experimental groups, compared with that in sham group. Administration of NaHS significantly augmented LC3 conversion, whereas the administration of PAG significantly dwindled LC3 conversion, compared with that in AP group. *In vitro*, there were three time‐points (1, 3 and 6 hrs) that we chose for observation (Fig. 2D, Fig. S2A and B). Generally, the duplicate results that resembled those at 6 hrs since AP induction *in vivo* were merely obtained when AP induction was terminated at 3 hrs *in vitro* (Fig. 2D). Thus, 3 hrs was taken as the end‐point for observation *in vitro*. Taken together, autophagic vacuoles were increased after AP induction and the extent of this increase was positively correlated with the incubated level of H_2_S.

### Trypsinogen pre‐mature activation positively correlated with the incubated level of H_2_S

**Figure 3:**
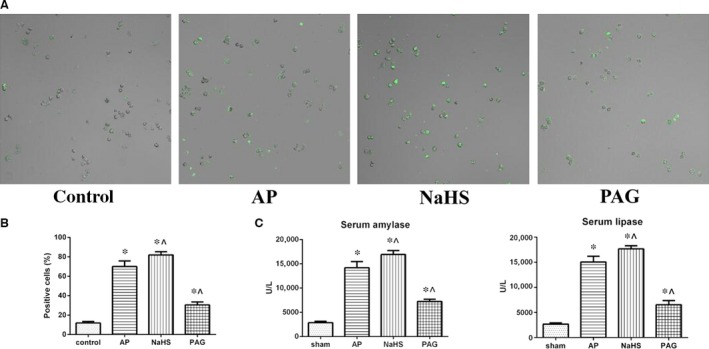
Trypsinogen pre‐mature activation positively correlated with the incubated levels of H_2_S. (**A**) Representative fluorescent photographs (10 × ) of trypsinogen activation assay in AR42J cells that were subjected to control, AP, AP+NaHS or AP+PAG for 40 min. since AP induction. (**B**) The percentage of positive (green) cells was calculated to quantify the extent of trypsinogen activation. (**C**) The serum levels of amylase and lipase in the peripheral blood samples harvested from the rats as described in Figure 1A were spectrophotometrically measured. Data were presented as mean ± S.D. (*n* = 3). *P < 0.05 *versus* sham or control, ^*P* < 0.05 *versus* AP.

We probed trypsinogen activation within AR42J cells using BZiPAR, a substrate for trypsin that becomes fluorescent after being cleaved by protease. As shown in Figure 3A and B, trypsinogen activation was significantly enhanced after AP induction when comparing the percentage of positive cells between control group and each of the experimental groups. Treatment with NaHS significantly enhanced the trypsinogen activation caused by AP induction alone, whereas treatment with PAG significantly reversed the trypsinogen activation caused by AP induction alone. Such effects of H_2_S that exerted on trypsinogen activation during AP were also suggested in part by the measurements of serum amylase and lipase (Fig. 3C). These results suggested that the extent of trypsinogen activation during AP was positively correlated with the incubated level of H_2_S.

### Autophagosome–lysosome fusion was not hampered but increased and was resistant to treatment with NaHS or PAG

**Figure 4:**
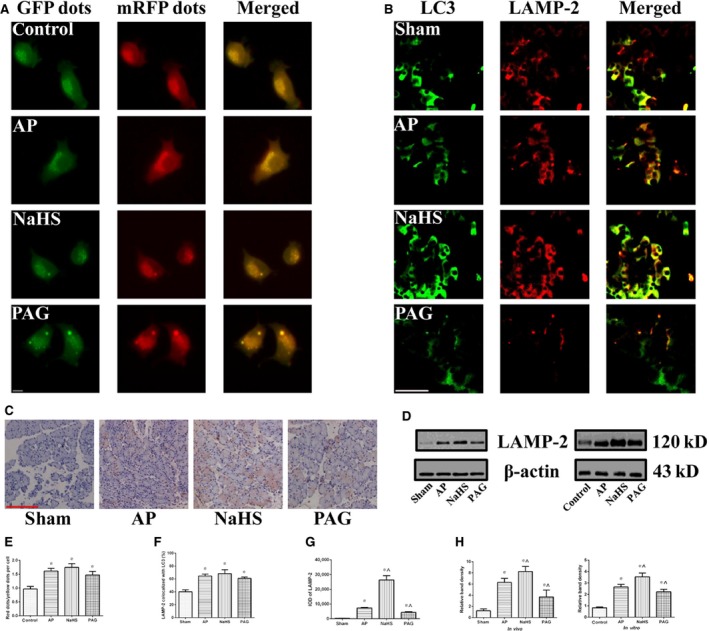
Autophagosome–lysosome fusion was not hampered but increased and was resistant to treatment with NaHS or PAG. (**A** and **E**) Representative fluorescent photographs (**A**) of autophagy flux assay in mRFP‐GFP‐LC3 tagged AR42J cells that were subjected to control, AP, AP+NaHS or AP+PAG for 3 hrs since AP induction. The ratio of red dots (autolysosomes) to yellow dots (autophagosomes) per cell (**E**) was calculated, bar: 50 μm. (**B** and **F**) Representative IF photographs (**B**) of colocalization between LAMP‐2 and LC3 in pancreatic tissues harvested from the rats as described in Figure 1A. The percentage of LAMP‐2 stained area that colocalized with LC3 (**F**) was evaluated, bar: 50 μm. (**C** and **G**) Representative IHC photograpgs (**C**) of LAMP‐2 expression in pancreatic tissues harvested from the rats as described in Figure 1A. The corresponding IODs (**G**) were analysed with Image‐Pro Plus v6.0 software, bar: 50 μm. (**D** and **H**) Representative immunoblot images (**D**) and quantitations (**H**) of LAMP‐2 expression in pancreatic tissues harvested from the rats (left) as described in Figure 1A and in AR42J cells (right) as described in Figure 2D. β‐actin was used as the protein loading control. Data were presented as mean ± S.D. (*n* = 3). *P < 0.05 *versus* sham or control, ^*P* < 0.05 *versus* AP.

An autophagy flux assay was performed with AR42J cells that were previously labelled with mRFP‐GFP‐LC3. As shown in Figure 4A and E, the ratio of red dots (autolysosomes) to yellow dots (autophagosomes) per cell was significantly increased in experimental groups when compared with that in control group indicating that the autophagosome–lysosome fusion was not hampered but increased after AP induction. Of note, there was no statistical significance between any two of the experimental groups, suggesting that the increased maturation of autophagosomes into autolysosomes after AP induction was immune to the further interventions against the incubated level of H_2_S. *In vivo*, an immunocolocalization assay between LAMP‐2 and LC3 was conducted. As shown in Figure 4B and F, the percentage of LAMP‐2 that colocalized with LC3 was significantly increased in experimental groups, compared with that in sham group. However, there was no difference regarding the percentage of LAMP‐2 that colocalized with LC3 between any two of experimental groups. So, the results of immunocolocalization assay between LAMP‐2 and LC3 *in vivo* agreed with that of autophagy flux assay *in vitro*. As an indispensable lysosome membrane protein for autophagosome–lysosome fusion 6, the LAMP‐2 expression was detected using IHC (Fig. 4C and G) afterwards. Integrated optical density was significantly increased after AP induction, and the administration of NaHS augmented the increase in IOD caused by AP induction alone; whereas, the administration of PAG significantly diminished the increase in IOD caused by AP induction alone. Figure 4D and H show the levels of LAMP‐2 expression detected using immunoblot both *in vivo* and *in vitro*. The level of LAMP‐2 expression was significantly increased in experimental groups, compared with that in sham/control group. Compared with that in AP group, the level of LAMP‐2 expression was significantly increased by the administration of NaHS but decreased by the administration of PAG. The results of immunoblot and IHC suggested that LAMP‐2 was up‐regulated in the AP model and its extent was positively correlated with the incubated level of H_2_S. Although LAMP‐2 is indispensable, its regulations by AP induction and treatments against incubated level of H_2_S are not directly associated with the genuine status of autophagosome–lysosome fusion. Therefore, based on the above‐mentioned results, it was rational to conclude that autophagosome–lysosome fusion was not hampered but increased in the Na‐TC‐induced AP model and was resistant to treatment with NaHS or PAG.

### Autophagy was over‐activated during AP and its extent positively correlated with the incubated level of H_2_S

**Figure 5:**
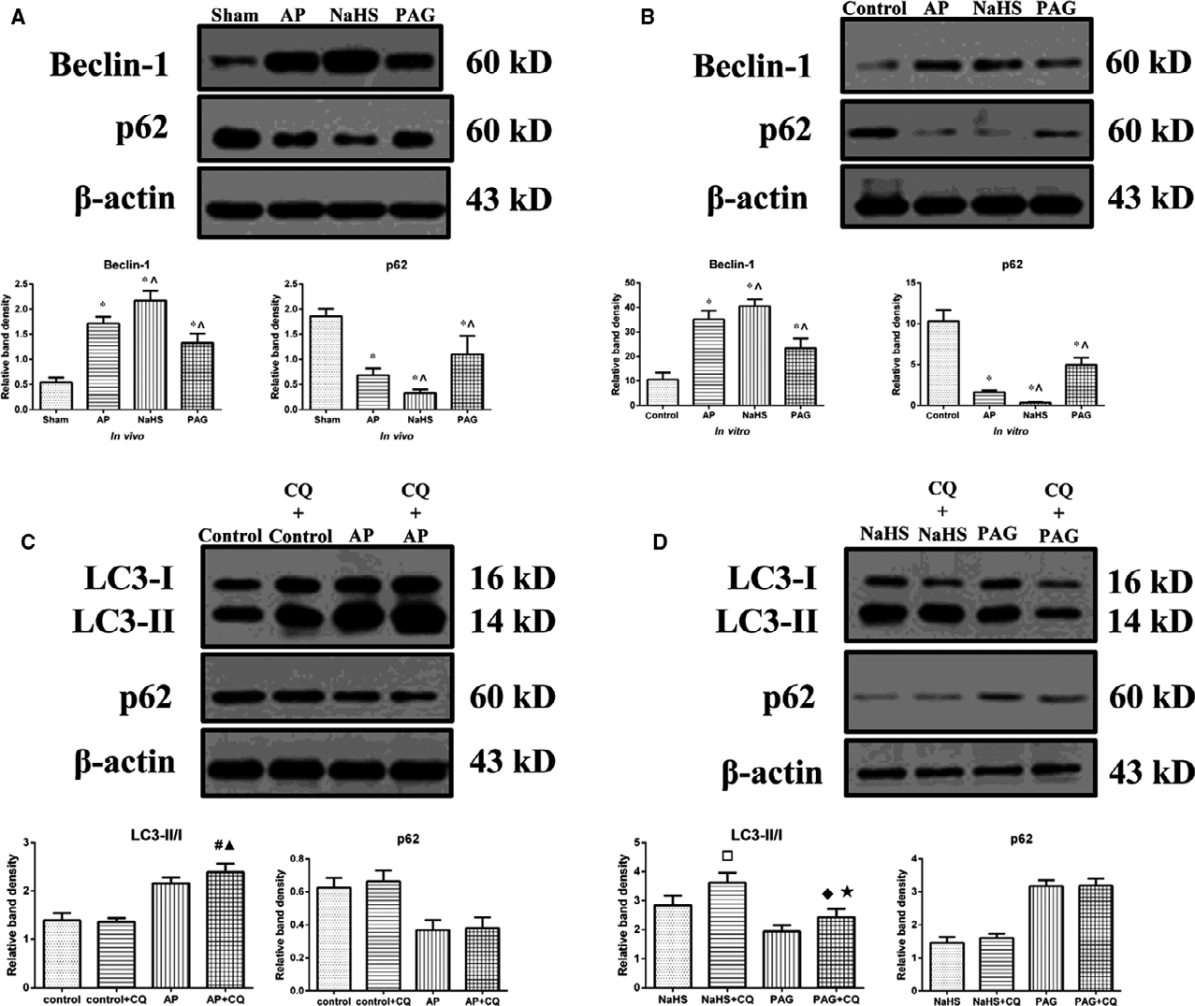
Autophagy was over‐activated during AP and the extent positively correlated with the incubated levels of H_2_S. (**A** and **B**) Representative immunoblot images (top) and quantitations (bottom) of both Beclin‐1 and p62 expressions in pancreatic tissues harvested from the rats (**A**) as described in Figure 1A and in AR42J cells (**B**) as described in Figure 2D. (**C**) Representative immunoblot images (top) and quantitations (bottom) of LC3 conversion and p62 expression in AR42J cells that were subjected to control or AP for 3 hrs since AP induction, with or without pre‐condition of CQ. (**D**) Representative immunoblot images (top) and quantitations (bottom) of LC3 conversion and p62 expression in AR42J cells that were subjected to AP+NaHS or AP+PAG for 3 hrs since AP induction, with or without pre‐condition of CQ. β‐actin was used as the protein loading control for all. Data were presented as mean ± S.D. (*n* = 3). *P < 0.05 *versus* sham or control, ^*P* < 0.05 *versus* AP, #*P* < 0.05 *versus* AP, ▲*P* < 0.05 *versus* control+CQ, □*P* < 0.05 *versus* NaHS, ♦*P* < 0.05 *versus* PAG, ★*P* < 0.05 *versus* NaHS+CQ.

To determine whether the autophagy was over‐activated, expressions of p62 (a substrate for autophagosome) and Beclin‐1 (an indicator of autophagy initiation) were detected by immunoblot. As shown in Figure 5A and B, after AP induction, the level of Beclin‐1 expression was significantly increased, whereas the level of p62 expression was significantly decreased, compared with that in control/sham group. In addition, the administration of NaHS significantly increased the level of Beclin‐1 expression and decreased the level of p62 expression, compared with that caused by AP induction alone. Regarding the administration of PAG, an opposite but still statistically significant finding was obtained: a decrease in the level of Beclin‐1 expression and a restore in the level of p62 expression. Furthermore, pre‐condition of CQ was administered to block lysosome‐driven degradation of autophagosomes *in vitro* and its efficacy was justified by a significant increase regarding LC3 conversion in the comparison of AP+CQ *versus* AP, NaHS+CQ *versus* NaHS and PAG+CQ *versus* PAG (Fig. 5C and D). With pre‐condition of CQ, AP induction was significantly associated with an increased LC3 conversion and a decreased level of p62 expression (control+CQ *versus* AP+CQ; Fig. 5C) indicating an up‐regulation in the upstream activity of autophagy process after AP induction. In the comparison between NaHS+CQ and PAG+CQ (Fig. 5D), treatment with NaHS was significantly associated with an increased LC conversion and a decreased level of p62 indicating that the extent of autophagy activation after AP induction were susceptible to the incubated level of H_2_S. Thus, all of the aforementioned findings suggested that H_2_S might be pro‐inflammatory during AP by over‐activating autophagy.

### H_2_S‐induced over‐activation of autophagy during AP was mediated by AMPK/mTOR pathway

**Figure 6:**
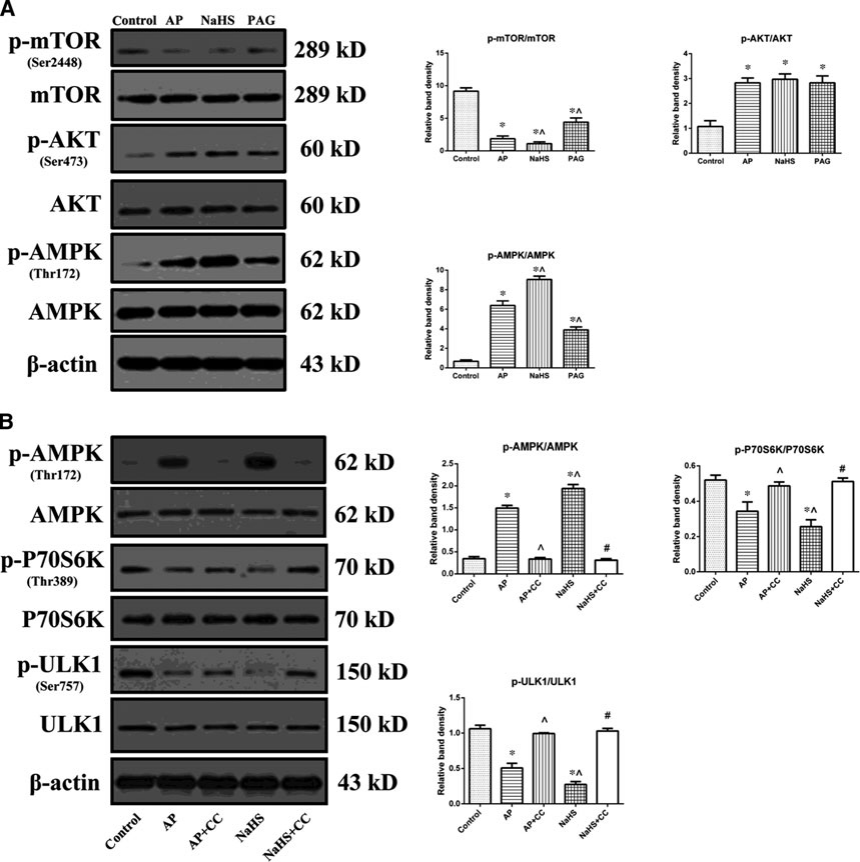
H_2_S‐induced over‐activation of autophagy during AP was mediated by AMPK/mTOR pathway. (**A**) Representative immunoblot images (left) and quantitations (right) of phosphorylations of AMPK, AKT and mTOR in AR42J cells as described in Figure 2D. (**B**) Representative immunoblot images (left) and quantitations (right) of phosphorylations of AMPK, P70S6k and ULK1 in AR42J cells that were subjected to control, AP, AP+CC, AP+NaHS or AP+NaHS+CC for 3 hrs since AP induction. β‐actin was used as the protein loading control for all. Data were presented as mean ± S.D. (*n* = 3). *P < 0.05 *versus* control, ^*P* < 0.05 *versus* AP, #*P* < 0.05 *versus* NaHS.

To elucidate the molecular mechanism that underlies H_2_S‐induced over‐activation of autophagy during AP, we performed immunoblot to examine phosphorylations of AMPK, AKT and mTOR *in vitro*. The ratio of p‐AKT/AKT was significantly increased in experimental groups compared with that in control group, but there was no difference regarding this index between any two of the experimental groups (Fig. 6A). On the other hand, the ratio of p‐AMPK/AMPK was significantly increased after AP induction while the ratio of p‐mTOR/mTOR was significantly decreased. Such alterations could be significantly potentiated by treatment with NaHS and restored by treatment with PAG.

To determine the involvement of mTOR signalling in H_2_S‐mediated over‐activation of autophagy during the pathogenesis of AP, CC (an inhibitor of AMPK) was introduced for pre‐treatment. Interestingly, not only the increased phosphorylation of AMPK, but also the decreased phosphorylations of P70S6K (a downstream substrate of mTOR Complex 1) and ULK1 (a downstream target of mTOR Complex 1) mediated by the administration of NaHS after AP induction were significantly suppressed by pre‐treatment of CC (Fig. 6B). Therefore, mTOR signalling acted as a downstream effecter of AMPK signalling in regulation of autophagy mediated by the administration of NaHS after AP induction.

## Discussion

Acute pancreatitis continues to be a clinical challenge, the pathogenesis of which remains elusive and for which no specific treatment has been developed so far 34, 35. Three death pathways might be evoked when the acini are doomed due to the attack of AP, namely necrosis, apoptosis and autophagy 36. To our knowledge, the interactions and switches among these cell death pathways could be triggered during AP and also could be regulated by various stimuli including H_2_S, which was investigated in our previous and present studies. It is becoming evident that H_2_S alters a large portion of mammalian proteins and their biological activity through sulphidation, a major post‐translational modification 37. Previously, our study suggested that H_2_S might be pro‐inflammatory during AP through inhibiting apoptosis and subsequently favouring a predominance of necrosis over apoptosis 14. Interestingly, the results of this study suggested that H_2_S exacerbated AP by over‐activating autophagy *via* AMPK/mTOR pathway, which represents another possible mechanism to interpret the detrimental effects of H_2_S during AP.

Two early‐phase characteristics within the pancreatic acini during AP have been long noticed, namely the accumulation of cytoplasmic vacuoles and the pre‐mature activation of trypsinogen. Recently, evidence has accumulated that both of these two pathological responses to AP could be attributed to the impaired autophagy 2, 3, 4, 5. Our findings demonstrated that both the accumulation of autophagic vacuoles (Fig. 2A) and the trypsinogen pre‐mature activation (Fig. 3A and B) were the most severe in NaHS group, followed by AP group and PAG group. Thus, we speculated that the detrimental effects of H_2_S during AP might be mediated by its regulation on the impaired autophagy.

Autophagy is a process by which damaged organelles and large molecules are destroyed, to relieve cellular stress and to provide nutrients to aid in cell survival. Accumulating evidence suggests that the impaired autophagy within acini is pivotal in the pathogenesis of AP. Theoretically, impaired autophagy could be attributed to two key sequential steps during the process, namely the formation of autophagosome that sequesters the organelles destined for degradation and the autophagosome–lysosome fusion to form autolysosome where materials are degraded by hydrolases 38. Hashimoto *et al*. 3 found that the Atg5 null mice were resistant to cerulein‐induced AP indicating that the formation of autophagosome might be significant in the development of the disease. Feng *et al*. 38 proposed that the protective effect of IL‐22 on cerulein‐induced AP in mice was mediated by inhibiting the formation of autophagosome. Similarly, TEM findings (Fig. 2A) suggested that the accumulation of autophagic vacuoles within acini during AP positively correlated with the incubated levels of H_2_S. Nevertheless, the accumulation of autophagic vacuoles could result from its excessive formation and/or inadequate clearance. The results indicated that the over‐activation of autophagy (Fig. 5) and not the hampered autophagosome–lysosome fusion (Fig. 4) might be suitable to answer the question. However, there are other opinions on this matter. Fortunato *et al*. 39 suggested that the impaired autophagy during AP might result from an attenuated autophagosome–lysosome fusion as demonstrated by LAMP‐2 depletion. The possible reasons for this deviation in this study are that: their AP model was induced by alcohol plus lipopolysaccharide (LPS) and the depletion of LAMP‐2 might result from the acute endotoxemia induced by LPS. In addition, the ultrastructural feature (a variety of partially degraded cargos) of autophagic vacuoles observed by TEM in the present study (Fig. 2A), was useful here to rule out the possibility of hampered autophagosome–lysosome fusion. Mareninova *et al*. 2 proposed that the impaired autophagy during AP was dominantly derived from a deficient lysosomal degradation due to the imbalance of cathepsin B and L, featuring AP as a lysosomal disease. We cannot deny this possibility in our models because a proper or even increased autophagosome–lysosome fusion does not mean an adequate clearance of autophagosomes. Although we did not conduct studies on the activities of cathepsins, this analysis on the upstream activity of autophagy by introducing CQ to block the lysosome‐driven degradation could be used at least to support that autophagy was activated during AP and the extent was positively correlated with the incubated level of H_2_S. The ratio of LC3II to LC3I, also named as LC3 conversion, indicates the net effects of formation and clearance of autophagosomes. The increased LC3 conversion after AP induction and its bilateral alterations by treatment with NaHS or PAG (Fig. 2C and D) could not be simply attributed to alterations on the formation of autophagosomes, although the detections of Beclin‐1 and p62 expression by immunoblot (Fig. 5A and B) suggested the significant alterations in the upstream activity of autophagy process. With pre‐treatment of CQ, the LC3 conversion analyses further confirmed the increased upstream activity of autophagy process after AP induction and its regulations by treatment with NaHS or PAG (AP+CQ *versus* control+CQ, NaHS+CQ *versus* PAG+CQ; Fig. 5C and D). Furthermore, the alterations of cytoplasmic HMGB1 expression (Fig. 1C), which were originally used to evaluate the necrotic injuries among the differently grouped pancreatic tissues, were also valuable here to support our findings. The translocation of HMGB1 from nuclei to cytoplasm facilitates the inhibition of Bcl‐2 by extracellular signal‐regulated kinase 1/2 and the binding of HMGB1‐Beclin‐1, therefore promoting the escape of Beclin‐1 from Bcl‐2 and subsequently facilitating phosphatidylinositol 3‐kinase‐Beclin‐1 complex assembly to initiate autophagy 40, 41.

AKT/mTOR pathway is well‐known for its regulation on autophagy 38, 42. In contrast to our expectations, the results (Fig. 6A) contradicted with the theory of AKT/mTOR pathway as suggested by the result that phosphorylation of AKT was not inhibited but enhanced after AP induction and was resistant to treatment with NaHS or PAG. Next we found that the mechanism which underlies the H_2_S‐induced over‐activation of autophagy during AP might be mediated by the activation of AMPK because AMPK was activated after AP induction and the extent was positively correlated with the incubated level of H_2_S (Fig. 6A). The rationale for this observation might lie in as follows: the increased ratio of AMP/ATP as a result of the attack of AP might stimulate AMPK, which is usually activated in response to a variety of extracellular and intracellular stresses 43, resulting in the activation of autophagy through the inhibition of mTOR. Given that the regulation of autophagy mediated by AMPK could be fulfilled without mTOR signalling 44, CC was introduced to determine the involvement of mTOR signalling in the model. We examined phosphorylations of P70S6K and ULK1. P70S6K, a downstream serine/threonine kinase of mTOR Complex 1, is involved in several cellular functions through phosphorylation mediated by activated mTOR 45. ULK1 (the mammalian homologue of the yeast Atg1), the most upstream component of core autophagy machinery, is also regulated by mTOR Complex 1 46. Besides the activation of AMPK, the down‐regulation of p‐P70S6k and p‐ULK1 that were mediated by the administration of NaHS in addition to AP induction were suppressed by pre‐treatment of CC (Fig. 6B), suggesting the involvement of mTOR signalling as a link between AMPK signalling and autophagy process. Thus, the molecular mechanism that underlies H_2_S‐mediated over‐activation of autophagy in AP consists of the activation of AMPK and the inhibition of mTOR, which is also referred to as AMPK/mTOR pathway. In addition, it was observed that phosphorylation of ULK1 at Ser757 was significantly decreased by the administration of NaHS than that induced by AP induction alone. As an inhibitory site regulated by mTOR, the relief of ULK1 Ser757 leads to autophagy induction through ULK1‐AMPK interaction and finally results in a vicious circle between decreased phosphorylation of ULK1 at Ser757 and increased activation of AMPK 47. It is another observation that supports our findings, that is, the impaired autophagy mediated by H_2_S in the pathogenesis of AP can be attributed, at least in part, to its excessive activation and initiation.

In spite of excellent researches done by various groups, the unravelling of H_2_S potentials in the pathogenesis of AP is far from over. Depending on dosage, donor type, exposure duration and other experimental conditions, H_2_S has shown either pro‐ or anti‐inflammatory properties 14, 15, 48. Generally, the physiological level of H_2_S might be cytoprotective, whereas the extra‐physiological level of H_2_S is considered to be toxic 12. Both our previous and present study suggested that the treatment with exogenous H_2_S donor exacerbated AP‐related injuries, which could be inversely attenuated by the treatment with endogenous H_2_S synthesizing inhibitor. Nonetheless, the debate regarding the biphasic effects of H_2_S during AP will continue because the mechanisms involved remain to be elucidated, along with the complicated interactions of the three known endogenously produced gaseous signalling molecules 49, 50.

In conclusion, H_2_S remains a pro‐inflammatory agent in the pathogenesis of AP. In the present study, we have, for the first time to our best of knowledge, questioned the detrimental role of H_2_S with specific respect to its regulation upon the impaired autophagy during AP. Our results suggested that H_2_S exacerbated AP by over‐activating autophagy *via* AMPK/mTOR pathway. Therefore, an active suppression of H_2_S might be a promising therapeutic approach against AP‐related injuries, and more studies regarding the actions and interactions of H_2_S in biological systems during AP are needed.

## Conflict of interest

The authors confirm that there are no conflicts of interest.

## Author contribution

LJ conducted most of the experiments and wrote the first draft. LL conducted the statistical analyses. FZQ and GQZ conducted the immunoblot *in vivo*. YWW and XWB provided technical assistance and contributed to the preparation of the figures. SHP provided technical assistance in IHC and haematoxylin‐eosin staining. DBX conducted the experiments shown in Figure 3. GW and BS conceived the idea for the project and revised the first draft. All authors reviewed the results and approved the final version of the manuscript.

## Supporting information


**Figure S1** Acute pancreatitis (AP)‐related pancreatic injuries (3 and 12 hrs), serum H_2_S levels and H_2_S synthesizing activities in pancreas (6 hrs).

**Figure S2** LC3 conversion at 1 and 6 hrs since AP induction *in vitro*.
