# Supplementary figures and images for: Hydrogen sulphide exacerbates acute pancreatitis by over‐activating autophagy via AMPK/mTOR pathway
Source: J Cell Mol Med. 2016 Jul 15;20(12):2349–61. doi: 10.1111/jcmm.12928 (PMC5134374; doi:10.1111/jcmm.12928)

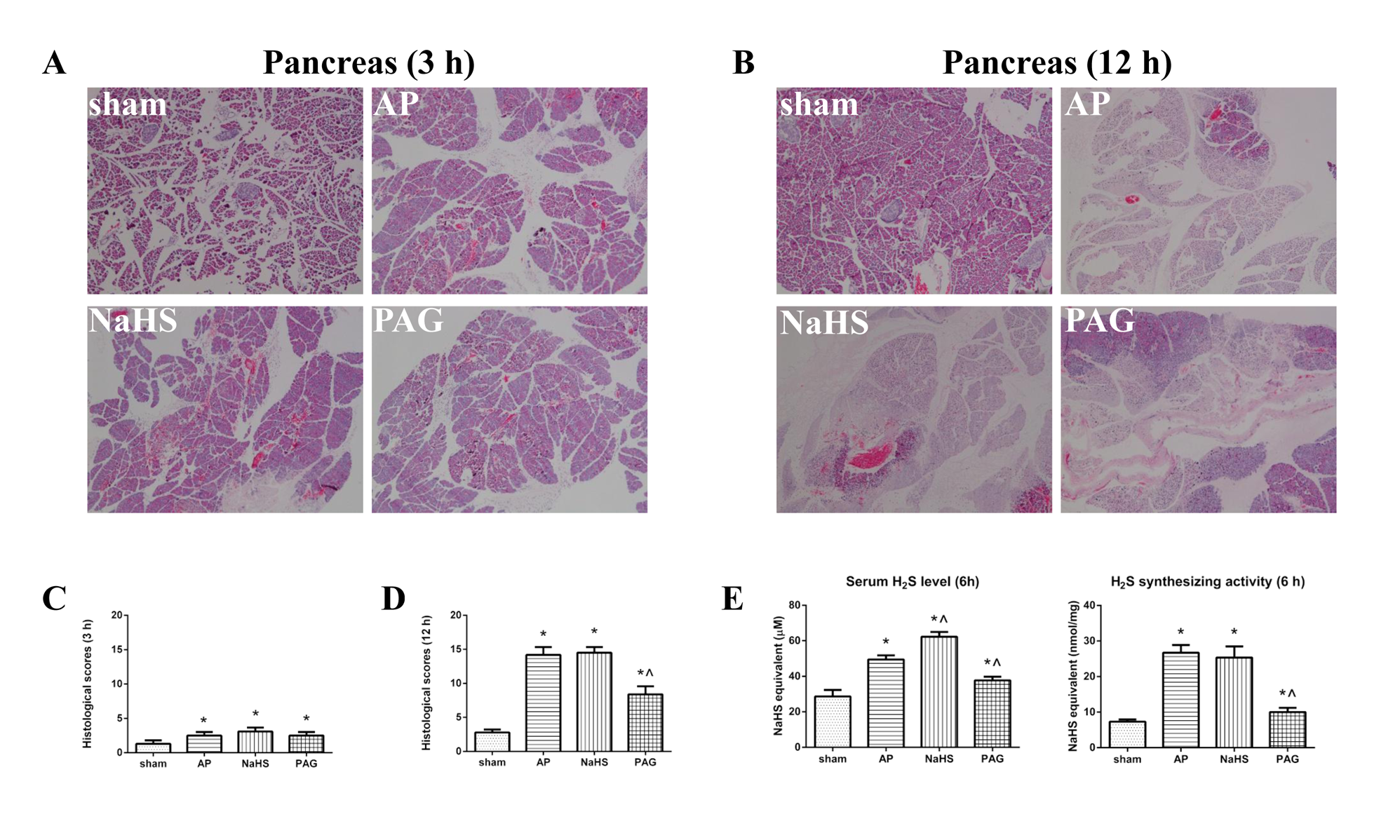

Supplement: Supplementary file 1 — Figure S1 Acute pancreatitis (AP)‐related pancreatic injuries (3 and 12 hrs), serum H2S levels and H2S synthesizing activities in pancreas (6 hrs). [file JCMM-20-2349-s001.tif]

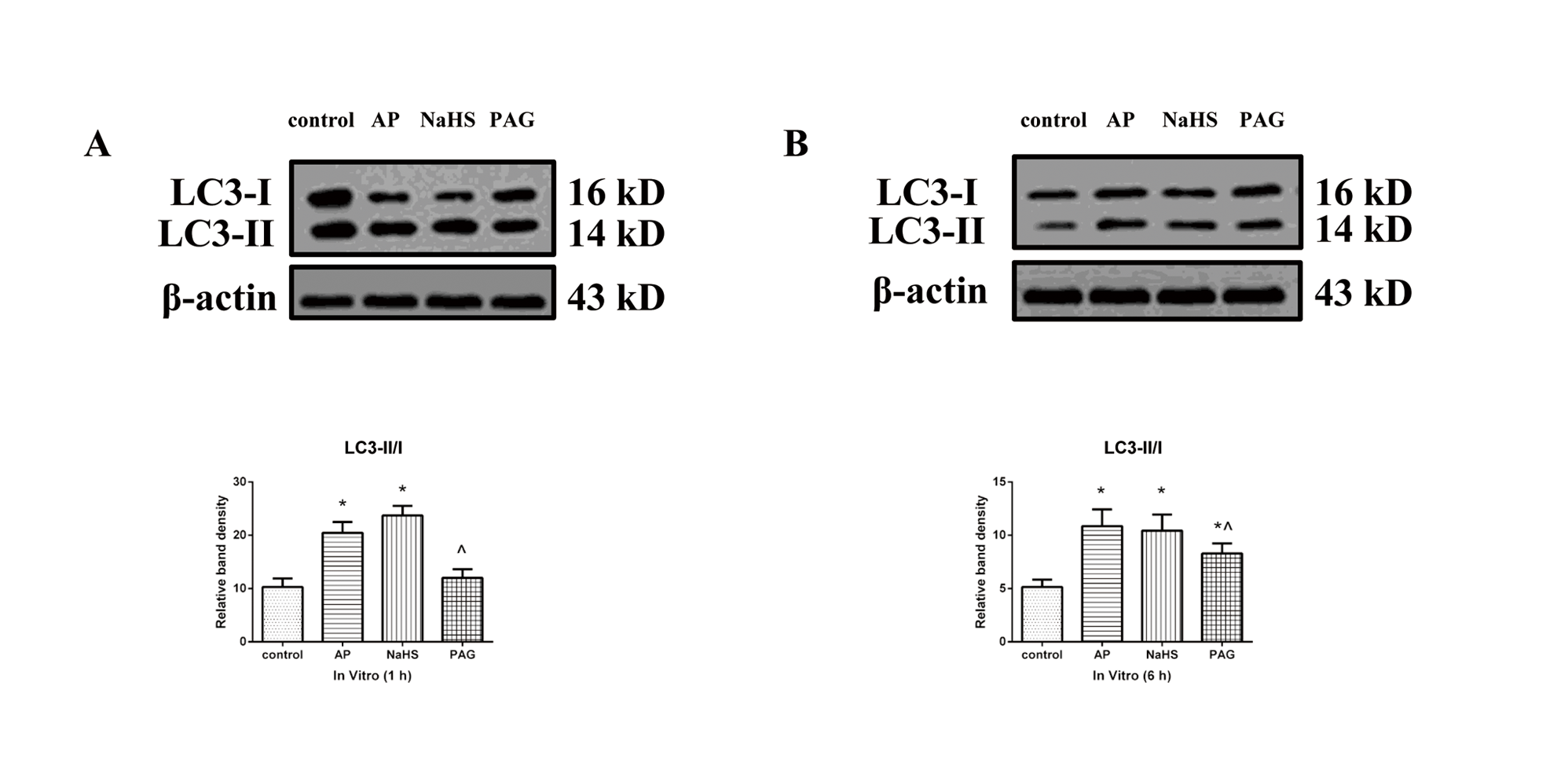

Supplement: Supplementary file 2 — Figure S2 LC3 conversion at 1 and 6 hrs since AP induction in vitro. [file JCMM-20-2349-s002.tif]
