# Supplementary material for: Hydrogen sulphide exacerbates acute pancreatitis by over‐activating autophagy via AMPK/mTOR pathway
Source: J Cell Mol Med. 2016 Jul 15;20(12):2349–61. doi: 10.1111/jcmm.12928 (PMC5134374; doi:10.1111/jcmm.12928)
Supplement: Supplementary file 3 [file JCMM-20-2349-s003.docx]

**Supplemental Fig. 1** AP-related pancreatic injuries (3 h and 12 h), serum H_2_S levels and H_2_S synthesizing activities in pancreas (6 h) (A, C) Representative photographs (A; 4×) and corresponding histopathological scores (C) of H&E-stained pancreatic tissues harvested from the rats that were subjected to sham operation, AP, AP+NaHS or AP+PAG for 3 h since AP induction. (B, D) Representative photographs (B; 4×) and corresponding histopathological scores (D) of H&E-stained pancreatic tissues harvested from the rats that were subjected to sham operation, AP, AP+NaHS or AP+PAG for 12 h since AP induction. (E) The serum H_2_S levels and H_2_S synthesizing activities in pancreatic tissues harvested from the rats as described in Fig. 1 A were calculated against a calibration curve of NaHS and expressed as NaHS equivalent. Data were presented as mean$\pm$SD (n=3). **p*<0.05 vs. sham, ^*p*<0.05 vs. AP.

**Supplemental Fig. 2** LC3 conversion at 1 h and 6 h since AP induction *in vitro* (A, B) Representative immunoblot images (top) and quantitations (bottom) of LC3 conversion in AR42J cells that were subjected to control, AP, AP+NaHS or AP+PAG for 1 h (A) and 6 h (B) since AP induction. β-actin was used as the protein loading control. Data were presented as mean$\pm$SD (n=3). **p*<0.05 vs. control, ^*p*<0.05 vs. AP.
